# Vitamines: A Brief Summary with a Forecast

**Published:** 1919-06-21

**Authors:** 


### VITAMINES.

### A Brief Summary with a Forecast.

THE term "vitamine" has now become so common in not only strictly medical literature, where the trained physician will understand the finer shades of its meaning, but also in the books and lectures of every health and welfare worker, with whom appreciation may not be so exact, that the recent explanatory letter by Professor Halliburton, comes most opportunely at a time when public-health proposals are so greatly to the fore, with such bright promise of achievement and yet in danger of error from over-confidence in theories whose youth may be their only claim to notice.

Elementary physiological teaching tells us that the human body is essentially built up from proteins, fats, carbohydrates, certain mineral salts, and water. Bodily energy causes a wastage of these substances, and a sufficiency of them must be ingested as food to repair the loss. Recently it has been established that alone these substances are not capable of maintaining healthy nutrition, and although the exact chemical and physical nature of the indispensable accessory food substances is unknown, the term chosen to denote them, in type at least, has been "vitamines."

The author points out that the simile of Professor Hopkins, who compares the relationship of these substances of the constituents of the body with that of mortar to the component bricks of a house, is correct, in the quantitative sense at least: "The mortar in the walls of a house makes up but a small proportion of the structure; it is exactly the same in the case of vitamines—they bear but a small proportion of the food supply." In spite of this, they are absolutely essential to healthy life and development, and although the practical meaning and perspective of these facts are very well known to every modern student of physiology, it is possible that the extraordinary power possessed by this class of substance is not fully appreciated by the average practitioner. He may know of all these "theories," but does he realise that, whatever the vital property may be, it is markedly lacking in so many of the so-called purified, prepared, and treated foods at present on the market? Almost without exception these foods are poor substitutes for Nature's own wares, and we know that animals are apparently unable to synthesize vitamines within their own bodies. These substances "are products of the plant world, and it is upon plants that all animals ultimately live."

Although three main groups, as described later, have been separated and classified under the somewhat non-committal terms introduced by American workers, there seems every likelihood that these form but the outposts of an army of factors soon to be disclosed as the cause of deficiency disease. In fact it is doubtful if any approach to realisation has yet been reached of the number of obscure, often trivial complaints which may ultimately come under this category. Many minor pathological states in early days tend to produce far-reaching results in later life, though at the time they may attract but slight attention. Professor Halliburton aptly cites the instance of dental caries, which, as Mrs. Mellanby has shown, is more than probably caused by a deficiency of a particular vitamine earlier in life—possibly "fat soluble A."

But the mention of this name brings one back to our classification. To take the first member, it is contained in the substance of the embryo in cereal seeds. In preparation, if milling is carried to a high degree, this part of the grain is almost entirely removed, so that although the finished products may appear ideal to the consumer, they are absolutely inferior as foods. Beri-beri is now definitely proved to be a deficiency disease, due to the continued use of polished rice, and both preventable and curable by adding the polishings to the diet. On account of its solubility in water it is termed, "water soluble B."

The second is found in the majority of animal fats in relatively high proportion, but is especially plentiful in milk fat and in cod-liver oil. Its particular utility in early life is due to its importance as a growth factor, and with reference to the general vegetable origin of these substances it is noteworthy that the green portions of plants contain this member in high concentration. In vegetable fats it is, however, practically absent, and "here we have one more indication of the value of milk for the young, an explanation of the potency of cod-liver oil in curing malnutrition, and a warning of the danger of vegetable margarines if employed as the only source of fat in the food of a growing section of the population or of expectant mothers." Probably this substance is an essential factor in rickets, and according to its solubility it is named "fat soluble A."

The third, or antiscorbutic factor, is termed "water soluble C," and is well known as contained in vegetable and fruit juices. Unfortunately, it is, we are told, destroyed by moderately high temperatures, alkalies, desiccation, canning processes, etc., and therefore the necessity that these food adjuncts should be fresh is obvious.

Professor Halliburton does not, however, aim in his article to indicate to the practitioner the elementary principles of the vitamine theory, although a thorough appreciation of advances in this direction is essential to every progressive worker for the nation's health. The point is that it is most unlikely that the present list of three vitamines represents more than a fraction of the total of such factors necessary to perfect nutrition. It is more than probable that very many minor but obscure complaints may ultimately be placed among the deficiency diseases, and of these we at least know their far-reaching effects, if not their exact causes. In the article is mentioned the valuable paper by Lieut.-Colonel R. McCarrison in a current volume of the *Indian Journal of Medical Research* on

**Vitamines**—*(continued)*.

"The Pathogenesis of Deficiency Disease," in which, although beri-beri is the disease particularly considered, it is shown that absence of "water soluble B" not only produces the typical neuritic symptoms of the condition, but also that every organ in the body is progressively affected and chronic inanition produced. At the same time certain organs, producing internal secretions, hypertrophy, while others atrophy in progressive order of severity. In February last was published a list of the author's conclusions, and perhaps the chief point to note is that there is more than a little evidence of interrelation between our vitamines and the internal secretions of the body. It may be that some of these substances are identical, or nearly so, but in any case one would point out to those for whom time does not allow of more than a superficial knowledge of these matters that, definite though they are, our three known vitamines are probably but the first signs of a new era in dietic and nutritional medicine. There is work galore to be done in this wide but fruitful field, and one must acknowledge the value and grace of the words in which Professor Halliburton has drawn attention to it.

